# Paediatric rheumatologic emergencies: clinical spectrum and outcomes from a 3-year cohort study

**DOI:** 10.1007/s00431-026-06829-2

**Published:** 2026-03-31

**Authors:** Jitendra S. Oswal, Vaibhav S. Bellary, Nikhila B. Kadimisetty, Bhakti Sarangi, Sushma Shree B C, Karthik Badarayan V

**Affiliations:** 1 Department of Clinical Immunology and Rheumatology, Bharati Vidyapeeth (Deemed to Be University) Medical College and Hospital, Pune-Satara Road, Bharati Vidyapeeth Campus, Dhankawadi, Pune, Maharashtra 411043 India; 2 Department of Paediatrics, Bharati Vidyapeeth (Deemed to Be University) Medical College and Hospital, Pune, Maharashtra India

**Keywords:** Macrophage activation syndrome, Systemic lupus erythematosus, ANCA vasculitis, Posterior reversible encephalopathy syndrome, Diffuse alveolar hemorrhage, Multisystem disease

## Abstract

Paediatric rheumatologic emergencies are rare but potentially fatal, and may present as the first manifestation of an underlying rheumatic disease. These conditions frequently present with nonspecific constitutional or organ-specific symptoms, posing a diagnostic challenge—particularly in children without a prior diagnosis. The objectives of this study were to evaluate the clinical characteristics and outcomes of paediatric rheumatological emergencies, comparing patients with and without known pre-existing rheumatologic conditions, and secondarily to suggest warning signs that may aid in the recognition of these situations. We conducted a retrospective cohort study over three-years at a tertiary paediatric rheumatology centre in western India. Medical records of all children (<18 years) diagnosed with rheumatologic diseases were reviewed. Demographic, clinical, and outcome data were analysed. Emergencies were defined as life-threatening or organ-threatening events requiring urgent evaluation or intensive care. Descriptive and comparative analyses were performed to evaluate outcomes between first-presentation and known rheumatic disease groups. Of 420 patients screened, 41 (9.8%) experienced rheumatologic emergencies; 30 (73.2%) presented as the first manifestation of disease. Common symptoms included fever (58.5%), dyspnea (46.3%), and seizures (17.1%). Major organ involvement included cardiovascular (31.7%), hematologic (26.8%), and central nervous (19.5%) systems. The most frequent acute emergencies were Macrophage Activation Syndrome (MAS) (23.4%), heart failure (19.15%), and respiratory failure (17.02%). Systemic Lupus Erythematosus (SLE) (51.06%) was the most common final diagnosis. Overall, 87.8% of patients recovered, while 12.2% succumbed, predominantly due to diffuse alveolar haemorrhage (DAH) and MAS.

*Conclusion*: Paediatric rheumatic diseases can present as life-threatening emergencies, frequently as the first manifestation of illness. Systemic lupus erythematosus was the most common underlying diagnosis in our cohort. Early recognition and timely initiation of immunosuppression may improve outcomes.

**Table:** 

**What is Known:** • *Paediatric rheumatologic diseases can rarely present as acute, life-threatening emergencies. Their nonspecific symptoms often mimic infections or malignancy, leading to diagnostic delays and preventable mortality, particularly in low-resource settings with limited rheumatology expertise.*
**What is New:** *This study characterizes real-world pediatric rheumatologic emergencies and provides practical red-flag indicators for early recognition and timely immunosuppression to improve survival in resource-limited settings*.

**What is Known:**

• *Paediatric rheumatologic diseases can rarely present as acute, life-threatening emergencies. Their nonspecific symptoms often mimic infections or malignancy, leading to diagnostic delays and preventable mortality, particularly in low-resource settings with limited rheumatology expertise.*

**What is New:**

*This study characterizes real-world pediatric rheumatologic emergencies and provides practical red-flag indicators for early recognition and timely immunosuppression to improve survival in resource-limited settings*.

## Introduction

Paediatric rheumatologic emergencies, though relatively uncommon, represent a critical and life-threatening subset of autoimmune conditions requiring urgent recognition and intervention [1]. These emergencies may present as the initial manifestation of an underlying systemic autoimmune disease. The challenge for paediatricians lies in the often nonspecific and subtle constitutional symptoms—such as fever, fatigue, or isolated organ dysfunction—which can be easily misattributed to infectious, hematologic, or oncologic causes, especially in children with no established rheumatic disease [2].

The rapid progression of such emergencies may result in catastrophic outcomes if not promptly identified and managed. Conditions such as macrophage activation syndrome (MAS), pulmonary-renal syndrome, central nervous system vasculitis, posterior reversible encephalopathy syndrome (PRES), and diffuse alveolar haemorrhage (DAH) can lead to high mortality [3]. Reported fatality rates for these emergencies can be as high as 50%, even with timely intervention [4]. In resource-limited settings, delays in recognizing autoimmune etiologies further increase the risk of misdiagnosis and inappropriate treatment.

Although literature on individual rheumatologic emergencies exists, systematic data on the spectrum of acute presentations in children who are eventually diagnosed with rheumatologic diseases remain sparse. Most evidence is limited to isolated case reports and small case series, highlighting the need for broader clinical characterizations. A better understanding of these serious clinical presentations can guide early evaluation, facilitate timely immunosuppressive therapy, and improve survival. Beyond describing their frequency and clinical spectrum, this study aims to identify key warning features that can help frontline paediatricians recognize these emergencies early and initiate appropriate immunosuppressive therapy before irreversible organ damage occurs.

## Objectives

The objective of our study was to evaluate the clinical characteristics and outcomes of paediatric rheumatological emergencies, comparing patients with and without known pre-existing rheumatologic conditions, and secondarily to suggest warning signs that may aid in the recognition of these situations.

## Methods

### Study design and setting

We conducted a retrospective cohort study at a tertiary-care centre in western India. The study included patients evaluated in the paediatric rheumatology inpatient and emergency services department over a three-year period from July 2022 to June 2025. This study is reported in accordance with STROBE guidelines [5].

### Study population

All paediatric patients (aged < 18 years) diagnosed with a rheumatologic disease during the study period were screened. Patients were eligible for inclusion if they had one or more life-threatening or severe systemic illnesses at presentation—such as shock, multiorgan dysfunction, acute respiratory distress, severe cytopenias or acute neurological deterioration. This included both patients already under rheumatology follow-up who later developed an emergency, as well as children referred to paediatric emergency or intensive care services and subsequently diagnosed with a rheumatologic disease.

Emergency presentation was defined as any acute, organ-threatening clinical event requiring urgent medical evaluation or hospitalization attributable to rheumatologic disease, including hematologic (e.g., MAS), neurologic (e.g., PRES, central nervous system [CNS] vasculitis), respiratory (e.g., respiratory failure, DAH), cardiovascular (e.g., heart failure, tamponade), renal (acute kidney injury), and gastrointestinal emergencies (e.g., lupus enteritis, pancreatitis) [6]. MAS was defined using established modified MAS criteria [7]. PRES was defined by characteristic clinical features and MRI findings consistent with posterior reversible encephalopathy [8]. DAH was defined by new pulmonary infiltrates with hemoptysis, falling hemoglobin and/or bronchoalveolar lavage showing red blood cells or hemosiderin-laden macrophages [9]. Acute kidney injury (AKI) was defined by Kidney Disease: Improving Global Outcomes (KDIGO) paediatric criteria [10]. Lupus enteritis was defined as either vasculitis or inflammation of the small bowel wall that is supported by either imaging or biopsy findings [11].

### Data collection

Demographic, clinical, and outcome data were extracted from medical records. Variables collected included age, sex, initial presenting features, emergency diagnosis, organ system involvement, final rheumatologic diagnosis, therapeutic interventions and outcomes. Information on any subsequent emergency episodes occurring during follow-up was also recorded, including the time interval, organ system(s) involved, and final outcome. Clinical manifestations were categorized as per organ involvement into cardiovascular, hematologic, neurologic, pulmonary, renal, and gastrointestinal systems.

*Outcome* was recorded as either:

- *Recovered:* Child survived and was clinically stable at discharge
- *Death:* Child succumbed to illness during the emergency admission

The treatment details were recorded as per clinical documentation in individual case records. Patients received immunosuppressive therapies based on clinical judgment at the time of presentation. These included glucocorticoids, cyclophosphamide, intravenous immunoglobulin (IVIg), disease-modifying antirheumatic drugs (DMARDs) and biologics (such as tocilizumab and rituximab) depending on the underlying diagnosis and severity of organ involvement. Emergency manifestations were recorded individually, and patients could contribute more than one manifestation per emergency episode when clinically applicable (e.g., DAH with concurrent AKI in Antineutrophil Cytoplasmic Antibody [ANCA] associated vasculitis). Therefore, the total number of manifestations exceeds the number of patients and episodes.

### Statistical analysis

Descriptive statistics were used to summarize baseline characteristics, clinical features, and outcomes. Categorical variables were expressed as frequencies and percentages. Continuous variables were presented as mean ± standard deviation (SD) or median with interquartile range (IQR), depending on distribution. Comparative analyses were performed between children presenting with a first-time rheumatologic emergency and those with a known rheumatologic disease: Categorical variables were compared using Fisher’s exact test. Continuous variables were compared using the Mann–Whitney U test. All analyses were conducted using International Business Machines Statistical Package for Social Sciences (IBM SPSS) Statistics, Version 29.0 (IBM Corp., Armonk, NY, USA). A *p*-value < 0.05 was considered statistically significant.

## Results

A total of 420 paediatric rheumatology patient records were screened over the three-year retrospective study period. A total of 47 emergency events were recorded in 41 patients. Among the 41 paediatric patients included, 30 (73.2%) presented with a rheumatologic emergency as the first clinical manifestation, while 11 (26.8%) had a pre-existing rheumatologic disease. There was no significant difference in the interval from symptom onset to recognition of the rheumatologic process between children presenting with a first-time rheumatologic emergency and those with a previously known rheumatic disease (p = 0.90).

The cohort included 23 females (56.1%) and 18 males (43.9%). The median age at emergency presentation was 120 months (interquartile range [IQR] 84–168), with a mean ICU stay of 9.3 ± 10.3 days and mean total hospital stay of 13.2 ± 11.9 days as shown in Table 1.

**Table 1:** Baseline demographic and clinical characteristics of children presenting with rheumatologic emergencies (*n* = 41)

Variable	Value
Number of patients	41
Female: Male (%)	23: 18 (56: 44)
Median age at emergency (months)	120 (84–168)
First presentation as emergency (%)	30 (73.2%)
Known rheumatic disease (%)	11 (26.8%)
Most common presenting symptoms	Fever (58.5%), dyspnoea (46.3%), headache (21.9%), seizures (17.1%), abdominal pain (17.1%), vomiting (14.6%), bleeding manifestations (14.6%), chest pain (14.6%), decreased urine output (14.6%)
Organ involvement	Cardiovascular (31.7%), haematologic (26.8%), central nervous system (19.5%), respiratory (19.5%), renal (17.1%), gastrointestinal (12.2%)

The most common first emergencies were MAS in 11 (23.4%) patients and heart failure in 9 (19.15%), followed by respiratory failure in 8 (17.02%) and AKI in 7 (14.9%). Other emergencies included shock in 5 (10.64%), PRES in 4 (8.51%), mesenteric vasculitis in 4 (8.51%), stroke in 3 (6.38%), and DAH in 3 (6.38%). Less frequent events included CNS vasculitis, cardiac tamponade, constrictive pericarditis (each in 2 patients; 4.26%), and pancreatitis, neuropsychiatric SLE (NPSLE) (1 patient each; 2.13%) as seen in Table 2. These represent emergency manifestations rather than mutually exclusive diagnoses, and individual patients could contribute more than one manifestation within the same episode (e.g., AKI with DAH in ANCA associated vasculitis). Final rheumatologic diagnoses after comprehensive evaluation most commonly included SLE (51.06%, n = 24), followed by systemic JIA (14.4%, n = 6), IgA vasculitis (9.8%, n = 4), and ANCA-associated vasculitis (9.8%, n = 4). JDM was diagnosed in three patients (7.3%), Takayasu arteritis in two patients (4.9%) and single cases (2.4% each) included Kawasaki disease, STING-associated vasculopathy with onset in infancy, camptodactyly–arthropathy–coxa vara–pericarditis (CACP) syndrome, and single organ vasculitis.

**Table 2:** Distribution of emergency manifestations by final rheumatologic diagnosis

	*n* (%)	SLE	SJIA	IgA vasculitis	AAV	JDM	TAK	Single organ vasculitis	CACP	KD	SAVI
MAS	11 (23.40)	4	6	–	–	1	–	–			
Heart failure	9 (19.15)	5	1	–	1	–	–	–	2	–	
Respiratory failure	8 (17.02)	1	–	–	3	3	–	–		1	
AKI	7 (14.90)	3	–	–	4	–	–	–	–	–	
Shock	5 (10.64)	3	–	–	1	–	–	–	–	1	
PRES	4 (8.51)	2	–	–	–	–	2	–	–	–	
Mesenteric vasculitis	4 (8.51)	–	–	4	–	–	–	–	–	–	
DAH	3 (6.38)	–	–	–	3	–	–	–	–	–	
Stroke	3 (6.38)	3	–	–	–	–	–	–	–	–	
CNS vasculitis	2 (4.26)	1	–	–	–	–	–	1	–	–	
Cardiac tamponade	2 (4.26)	–	–	–	–	–	–	–	1	–	1
Constrictive pericarditis	2 (4.26)	–	1	–	–	–	–	–	1	–	–
NPSLE	1 (2.13)	1	–	–	–	–	–	–			
Pancreatitis	1 (2.13)	1	–	–	–	–	–	–			

Values represent number of patients with the specified manifestation within each diagnostic category. Emergency manifestations were not mutually exclusive; individual patients could present with multiple simultaneous organ-threatening features during the same emergency episode (e.g., acute kidney injury and diffuse alveolar haemorrhage  in AAV). Therefore, column totals exceed the number of patients

*Abbreviations*: *MAS* macrophage activation syndrome, *AKI* acute kidney injury, *PRES* posterior reversible encephalopathy syndrome, *DAH* diffuse alveolar haemorrhage, *CNS* central nervous system, *NPSLE* neuropsychiatric systemic lupus erythematosus, *SLE* systemic lupus erythematosus, *SJIA* systemic juvenile idiopathic arthritis, *AAV* ANCA-associated vasculitis, *JDM* juvenile dermatomyositis, *TAK* Takayasu arteritis, *KD* Kawasaki disease, *SAVI* STING-associated vasculopathy with onset in infancy

Overall outcomes were favourable in most patients, with 38 (92.68%) of 41 children showing recovery following appropriate immunosuppressive therapy during their first emergency episode. However, 3 (7.32%) children died during the initial emergency admission—two with ANCA-associated vasculitis died due to DAH, and one child with SLE due to acute renal failure complicated by shock.

During follow-up, 5 children (13.2%) experienced a second emergency episode, most commonly among those with SLE or vasculitis with major organ involvement. Of these, 4 recovered, and 1 patient died during the second episode due to MAS. Furthermore, 1 of the 4 recovered patients went on to have a third emergency, which also resulted in death. This child was eventually diagnosed with CACP syndrome who died due to refractory congestive cardiac failure. In total, 5 children (12.2%) died during the study period — 3 during the first emergency, 1 each during the second and third emergencies.

Comparative analyses between first-time and known disease presentations revealed no significant differences in mortality, recurrence, or hospitalization metrics (Table 3). These findings suggest that outcomes following rheumatologic emergencies were similar in children presenting de novo and those with pre-existing disease, although the study was not powered to detect small differences.

**Table 3:** Comparison of outcomes between first-presentation and known rheumatologic disease groups

Variable	First presentation (*n* = 30)	Known disease (*n* = 11)	*p*-value
Mortality (*n*)	3	0	0.55
Recurrence (*n*)	3	2	0.62
ICU stay, median (IQR), days	5.5 (3–9)	8 (5–11)	0.30
Total hospital stay, median (IQR), days	8 (5.2–15.8)	12 (7.5–13.5)	0.42
Recovery time, median (IQR), days	14 (9–28)	20 (13–24)	0.32

Values are expressed as median (interquartile range) for continuous variables and compared using the Mann–Whitney U test. Categorical variables are expressed as counts and compared using Fisher’s exact test

*ICU* intensive care unit, *IQR* interquartile range

## Discussion

This study highlights the critical need for heightened awareness among paediatricians and emergency physicians, as rheumatologic diseases frequently masquerade as common paediatric emergencies [12]. Strikingly, 73.2% of paediatric patients experienced their first-ever rheumatologic manifestation as an acute emergency, underscoring the diagnostic challenge these cases pose—especially in the absence of prior rheumatologic suspicion. In real-world practice, rheumatologic emergencies can mimic common paediatric illnesses —presenting with isolated fever, dyspnoea, pain abdomen, headache or seizures. Without appropriate clinical suspicion, such presentations are often misattributed to infectious, cardiac, or neurologic causes, leading to delayed diagnosis and misdirected interventions. The spectrum of emergency presentations and underlying rheumatic conditions reflects the wide heterogeneity and diagnostic complexity encountered in paediatric rheumatologic emergencies.

To guide early recognition and management at the point of first contact, we propose a practical red-flag framework for frontline paediatricians (Table 4). These clinical indicators, although not definitive, can assist paediatricians in differentiating rheumatologic emergencies from mimicking conditions and prompt early referral. However, clinical judgment and multidisciplinary evaluation remain essential, as rheumatic diseases in children often present atypically.

**Table 4:** Red flag signs for rheumatic diseases

Red flag signs	Possible emergency diagnosis	Action required
High-grade persistent fever + cytopenias	MAS	Evaluate ferritin, triglycerides, Fibrinogen, ESR, Liver enzymes. Urgent immunosuppression
Ferritin > 2000 ng/mL	MAS	Urgent immunosuppression (IV glucocorticoids ± cyclosporine)
Rapidly worsening effusions (pleural/pericardial)	SLE, MAS, vasculitis	Echocardiography, immunosuppressive therapy
Altered mental status, seizures, psychosis	Neuropsychiatric SLE, CNS vasculitis	MRI brain, CSF studies, immunosuppression
Purpura + hypertension + abdominal pain	IgA vasculitis, PAN	Monitor renal function, consider glucocorticoids
Unexplained hepatitis or transaminitis	MAS, SLE	Screen for MAS, autoimmune hepatitis
Unexplained acute renal failure	Lupus nephritis, vasculitis	Urinalysis, complement levels, renal biopsy (if stable)
Sudden sensorineural hearing loss or vision loss	Behçet’s disease, vasculitis	ENT or ophthalmologic evaluation, immunosuppression
Unexplained severe abdominal pain ± vomiting/diarrhoea	Lupus enteritis, Gastrointestinal vasculitis	Abdominal imaging, stool occult blood, immunosuppression, gastroenterologist consultation

*MAS* macrophage activation syndrome, *ESR* erythrocyte sedimentation rate, *IV* intravenous, *SLE* systemic lupus erythematosus, *CNS* central nervous system, *MRI* magnetic resonance imaging, *CSF* cerebrospinal fluid, *IgA* immunoglobulin A, *PAN* polyarteritis nodusa, *ENT* ear, nose and throat

MAS was the most common life-threatening emergency, present in 23.4% of first presentations. Its fulminant form occurs in ~ 10% of patients with SJIA, with subclinical forms affecting up to 30–40% [7, 13]. Mortality can be as high as 32% in Indian cohorts, often due to late-stage multiorgan dysfunction. Evidence shows that early initiation of cytokine-specific therapy (e.g., anakinra within 5 days) is associated with reduced MAS mortality [13, 14]. Neuropsychiatric lupus, including presentations with PRES, occurs in ~ 25% of children with SLE, but is often underdiagnosed [15]. These children may present with seizures, altered sensorium, or psychosis, which mimic CNS infections [16]. Pulmonary haemorrhage, although rare, carries a high mortality of up to 50% in paediatric SLE and 25–75% in vasculitis such as IgA vasculitis [6, 9, 16]. DAH was among the most fatal emergencies in our cohort, contributing to mortality in children with both lupus and vasculitis, consistent with prior reports [17]. In our series, such events often preceded a formal rheumatologic diagnosis, further highlighting the urgency of early recognition.

Comparative analysis between first-presentation and known disease groups showed no statistically significant differences in mortality, recurrence, or hospitalization parameters, suggesting that once a rheumatologic emergency develops, outcomes may be more strongly influenced by timely recognition and treatment than by prior diagnosis, although this observation should be interpreted cautiously given the small sample size. Encouragingly, these findings exist alongside a high recovery rate in our cohort—87.8% of patients ultimately survived and recovered—reinforcing the critical value of timely diagnosis and rapid immunosuppression. Delays in referral and lack of familiarity with red-flag symptoms in paediatric rheumatology remain major contributors to adverse outcomes.

Practical management priorities for frontline clinicians include:

- Do not delay immunosuppression if clinical suspicion for MAS, SLE or vasculitis is high
- Early pulse methylprednisolone may be life-saving in selected cases
- Even in the absence of a rheumatologist, consultation with them remotely is advised
- Establish follow-up protocols post-emergency to detect relapses early
- These emergencies require timely immunomodulatory treatment rather than cardiac or neurology super-specialists.

Our findings underscore several key imperatives to improve outcomes in paediatric rheumatologic emergencies:

- Training frontline paediatricians to identify atypical presentations and rheumatologic etiologies early.
- Promoting the use of paediatric emergency red-flag checklists in emergency departments [18].
- Establishing rapid referral pathways to tertiary care centres or leveraging tele-rheumatology support in settings lacking specialist availability [19].

This single-centre, real-world study reflects the clinical diversity and diagnostic challenges of paediatric rheumatologic emergencies. Limitations include its retrospective design, modest sample size, and limited event count, which precluded regression or survival analyses. Referral bias is also possible given the tertiary-care setting. Accordingly, observed associations should be interpreted as exploratory and hypothesis-generating rather than causal.

## Conclusion

Rheumatic diseases in children can present as life-threatening emergencies, often as the first manifestation of the underlying illness. In this cohort, systemic lupus erythematosus was the most common diagnosis, and nearly three-quarters of children presented without a prior rheumatologic diagnosis. Early clinical suspicion, recognition of red-flag features, and prompt initiation of immunosuppressive therapy are likely to be important in improving outcomes. Strengthening awareness among frontline paediatricians and establishing clear referral pathways may help reduce diagnostic delays and improve survival, particularly in resource-limited settings.

## Data Availability

The dataset has been uploaded in a public data repository (Figshare) and the link to access is [10.6084/m9.figshare.30229759.v1].

## Abbreviations

AKI: Acute kidney injury
APLA: Antiphospholipid antibody syndrome
ANA: Antinuclear antibody
ANCA: Antineutrophil cytoplasmic antibody
CACP: Camptodactyly–arthropathy–coxa vara–pericarditis
CNS: Central nervous system
DAH: Diffuse alveolar haemorrhage
DMARDs: Disease-modifying antirheumatic drugs
ICU: Intensive care unit
IL: Interleukin
IVIg: Intravenous immunoglobulin
JDM: Juvenile dermatomyositis
MAS: Macrophage activation syndrome
NPSLE: Neuropsychiatric systemic lupus erythematosus
PRES: Posterior reversible encephalopathy syndrome
PRS: Pulmonary–renal syndrome
SAVI: STING-associated vasculopathy with onset in infancy
SD: Standard deviation
SJIA: Systemic juvenile idiopathic arthritis
SLE: Systemic lupus erythematosus
STING: Stimulator of interferon genes
